# GNN-surv: Discrete-Time Survival Prediction Using Graph Neural Networks

**DOI:** 10.3390/bioengineering10091046

**Published:** 2023-09-06

**Authors:** So Yeon Kim

**Affiliations:** 1 Department of Artificial Intelligence, Ajou University, Suwon 16499, Republic of Korea; jebi1771@ajou.ac.kr; 2 Department of Software and Computer Engineering, Ajou University, Suwon 16499, Republic of Korea

**Keywords:** discrete survival model, Graph Neural Networks, patient similarity network, survival prediction, time-to-event prediction

## Abstract

Survival prediction models play a key role in patient prognosis and personalized treatment. However, their accuracy can be improved by incorporating patient similarity networks, which uncover complex data patterns. Our study uses Graph Neural Networks (GNNs) to enhance discrete-time survival predictions (GNN-surv) by leveraging relationships in these networks. We build these networks using cancer patients’ genomic and clinical data and train various GNN models on them, integrating Logistic Hazard and PMF survival models. GNN-surv models exhibit superior performance in survival prediction across two urologic cancer datasets, outperforming traditional MLP models. They maintain robustness and effectiveness under varying graph construction hyperparameter μ values, with performance boosts of up to 14.6% and 7.9% in the time-dependent concordance index and reductions in the integrated brier score of 26.7% and 24.1% in the BLCA and KIRC datasets, respectively. Notably, these models also maintain their effectiveness across three different types of GNN models, suggesting potential adaptability to other cancer datasets. The superior performance of our GNN-surv models underscores their wide applicability in the fields of oncology and personalized medicine, providing clinicians with a more accurate tool for patient prognosis and personalized treatment planning. Future studies can further optimize these models by incorporating other survival models or additional data modalities.

## 1. Introduction

The criticality of acknowledging censored observations in cancer research for accurate survival prediction is unquestionable [1]. Censored observations, such as patients lost to follow-up or outliving the study duration, often emerge in oncology studies. Overlooking these observations may cause significant bias in survival time and probability estimates, compromising the reliability of the study’s findings [2]. Proper handling of censoring in survival analysis, therefore, is a cornerstone of oncology research, allowing a comprehensive and accurate portrayal of patient survival patterns, with significant implications for prognosis and clinical decision making.

Survival analysis involves a broad spectrum of continuous- and discrete-time survival models. The Cox proportional hazards regression model [3], a well-regarded continuous-time survival model, offers flexibility and interpretability. It estimates the hazard function based on a baseline hazard and an exponential function of linear predictors. The random survival forest model [4], an extension of the random forest model for right-censored survival time data, employs a decision tree ensemble trained on bootstrap data samples for robust survival time predictions. In contrast, discrete-time survival models like logistic regression models analyze hazard rates in discrete-time intervals [5,6], particularly beneficial when exact event times are unknown.

Recently, there has been an upsurge in using deep learning and machine learning for cancer survival prediction [7,8,9,10,11,12,13,14,15,16,17]. These advanced techniques excel at handling high-dimensional and heterogeneous data, unveiling complex patterns that traditional statistical models may miss. However, most of these studies focus predominantly on classifying patients into long-term or short-term survival groups, simplifying the multifaceted reality of patient survival times and often neglecting censored data.

To address the challenges, many researchers have developed survival models that incorporate neural networks with survival analysis. Kvamme et al. [12] combined the neural networks and Cox regression, offering a robust method to analyze survival data. Similarly, DeepSurv [13] proposed a Cox proportional hazards deep neural network, a state-of-the-art survival method to model the interplay between patients’ covariates and treatment effectiveness to facilitate personalized treatment recommendations. Furthermore, DeepHit [14] introduced a deep neural network for survival analysis, specifically accounting for competing risks. This approach is particularly crucial for scenarios where multiple potential events of interest exist. In the domain of discrete-time survival models, a pioneering work by Brown [15] utilized indicator variables, which provided insights into how discrete markers can enhance survival model performance. Building on this, Nnet-survival [16] proposed a scalable approach to discrete-time survival modeling, which is designed to be used with neural networks. Collectively, these studies have advanced the integration of neural networks into survival analysis.

In the field of cancer research, Graph Neural Networks (GNNs) have achieved significant progress, uncovering intricate relationships often overlooked by traditional models. MGNN [18,19] provides a unified framework by building bipartite graphs between patients and multimodal data like gene expression and clinical information, demonstrating its efficacy in classifying short- and long-term survival across four cancer datasets. Qiu et al. [20] introduced an intratumor GNN model that leverages the spatial heterogeneity of multiple in situ biomarkers to reveal hidden prognostic value in breast cancer cases. The model’s prognostic capability rivals that of conventional methods using routine biomarkers, advancing cancer prognosis. PathGNN [21] proposed a GNN model that can capture topological features in cancer pathways to predict long-term survival, identifying critical pathways linked to cancer outcomes. These studies underscore the versatility and effectiveness of GNNs in a variety of oncological research contexts. However, they still face challenges in appropriately considering censoring status in survival models, affecting the reliability of survival predictions. Although numerous studies have applied GNNs in survival prediction, their use in censoring-aware survival models remains largely unexplored. To refine and enhance the reliability of survival predictions, it is essential to integrate GNNs with survival models capable of effectively managing censoring. Our experiments with GNNs for discrete-time survival prediction models demonstrate this assumption, outperforming models that do not account for these relational structures.

In this study, we propose the hypothesis that distinct patient groups, characterized by similar genomic and clinical features, significantly influence their survivability and mortality rates. Recognizing these intergroup correlations in survival prediction can substantially improve the performance. We assume that relational structures exist within cancer patient data, contributing considerably to accurate survival predictions. We adopt patient similarity networks and GNNs to comprehend complex correlational structures and propose a GNN model specifically designed for discrete-time survival prediction (GNN-surv). As a proof-of-concept study, we conducted experiments on bladder and kidney cancer datasets. Our experiments demonstrated the effectiveness of the GNN-surv models in predicting discretized survival times, thus validating our hypothesis and research motivation. Our findings further highlight the importance of addressing the question of censoring in real-world scenarios and the potential for the broad applicability of GNN-surv models across diverse cancer datasets. The main contributions are summarized as follows.

- We design and construct a sophisticated cancer patient similarity network that integrates both genomic and clinical features, enabling a better understanding of patient characteristics and relationships.
- We propose GNN-surv, a novel GNN that incorporates discrete-time survival models. We demonstrate its broad applicability via experiments across two different survival models and three types of GNN layers.
- We empirically show the superior performance of GNN-surv in survival prediction for two urologic cancers, thereby showing its potential for broader application in oncological research.

## 2. Materials and Methods

### 2.1. Dataset

We obtained the RNA-Seq gene expression profiles of the TCGA bladder cancer (BLCA) and kidney clear cell carcinoma (KIRC) datasets. The gene expression data, comprising estimates for 20,530 genes, were measured using Illumina HiSeq 2000 RNA Sequencing, a level 3 data source from the TCGA data coordination center. We note that the same number of genes, 20,530, was present in both the BLCA and KIRC datasets. We retrieved these log-transformed RSEM normalized count data from the UCSC Xena platform [22].

The datasets included 400 patients for BLCA and 313 patients for KIRC. We excluded any patients with unrecorded or inaccurate clinical outcome variables, such as negative survival day values. For both cancer datasets, we used clinical variables such as overall survival (OS), event status, age, gender, and TNM stage. The event status was binary, with 1 indicating that an event occurred and 0 representing right-censored cases. The BLCA dataset contained 223 censored and 173 uncensored samples, yielding a censoring rate of 56.3%, whereas the KIRC dataset comprised 209 censored and 102 uncensored samples, yielding a censoring rate of 67.2%. In this study, we selected two types of urologic cancer as our subjects to illustrate the proof-of-concept. Although the TCGA dataset provides data for six types of urologic cancers, we decided to exclude certain types due to specific reasons. Testicular cancer (TGCT) and kidney chromophobe (KICH) were excluded due to their small sample sizes of 134 and 65 cases, respectively. Similarly, the kidney papillary cell carcinoma (KIRP) and prostate cancer (PRAD) datasets were not utilized. Despite their adequate sample sizes, these datasets were ruled out due to the excessive censoring rates of 85.1% and 98.2%, respectively. Such high censoring rates could introduce bias into our prediction model, thereby compromising the model’s performance and the interpretation of the results [23].

We dichotomized the clinical variables, grouping ages into less than 65 years (0) and 65 years or older (1), T stages into T0-2 (0) and T3-4 (1), N stages into N0 and N1-3 (N+), and M stages into M0 and M1. Where pathologic stages were unknown, we filled in the gaps based on the American Joint Committee on Cancer (AJCC) staging system. Missing N or M stages were inferred from the number of positive lymph nodes or metastatic sites. For instance, cases with any positive lymph nodes were classified as N+, and if the only recorded metastatic site was ’lymph node only,’ it was classified as M0. Notably, metastatic site data were only available in the BLCA dataset. We adopted this preprocessing strategy for clinical variables from the study [24]. The summary statistics of clinical features for both datasets are displayed in Table 1.

### 2.2. Patient Similarity Graph

We construct a patient similarity graph, denoted as G=(V,E), where the vertex set $V=(v_{1},\ldots ,v_{n})$ symbolizes the cohort of cancer patients. Each patient, or vertex $v_{i}$, is characterized by a feature vector $x_{i}$, which combines their RNA-seq gene expression and clinical features. The set E defines undirected edges.

While clinical features are discretized into categories of 0 or 1, gene expression features are continuous variables. In joining genomic and clinical variables, we standardize gene expression features by subtracting the mean and scaling to unit variance.

We calculate patient similarity between patients $v_{i}$ and $v_{j}$ as W(i,j), derived from a scaled exponential similarity kernel, predicated on the probability density function of the normal distribution as follows:

(1) $$W\left(i,j\right)=\frac{1}{\sqrt{2\pi \sigma^{2}}}\exp-\frac{\rho^{2}\left(x_{i},x_{j}\right)}{2\sigma^{2}}$$

where $\rho (x_{i},x_{j})$ denotes the pairwise correlation distances between patients $x_{i}$ and $x_{j}$. We compute the value σ as follows:

(2) $$\sigma =\mu \frac{\bar{\rho }\left(x_{i},N_{i}\right)+\bar{\rho }\left(x_{j},N_{j}\right)+\rho \left(x_{i},x_{j}\right)}{3}$$

Here, $\bar{\rho }\left(x_{i},N_{i}\right)$ signifies the average value of the distances between $x_{i}$ and its neighbors $N_{1\ldots k}$, where *k* is the number of neighbors considered. μ is a hyperparameter modulating the extent to which we scale the similarity kernel *W*, and μ∈(0,1)⊂R.

We define the adjacency matrix A(i,j) as 1 if W(i,j)>c, where *c* is a correlation threshold, and otherwise A(i,j)=0.

This study relies heavily on graph structures; thus, we utilize a sophisticated patient similarity network construction method proposed by [25]. A study’s performance can be sensitive to the hyperparameter μ, also discussed in [25], leading us to empirically set μ in the range of [0.1, 1.0].

**Table 1:** Summary statistics of clinical features in the TCGA bladder cancer (BLCA) and kidney clear cell carcinoma (KIRC) data.

Feature		BLCA	KIRC
Number of Patients		400	313
Age	<65 years	147 (36.8%)	193 (61.7%)
	≥65 years	253 (63.2%)	120 (38.3%)
Gender	Male	295 (73.8%)	201 (64.2%)
	Female	105 (26.2%)	112 (35.8%)
Stage T (Primary tumor)	Negative (Stages T0–2)	148 (37.6%)	196 (62.6%)
	Positive (Stages T3–4)	246 (62.4%)	117 (37.4%)
Stage N (Regional lymph nodes)	Negative (Stage N0)	261 (67.4%)	244 (87.8%)
	Positive (Stage N1–3)	126 (32.6%)	34 (12.2%)
Stage M (Distant metastasis)	Negative (Stage M0)	340 (86.1%)	258 (82.7%)
	Positive (Stage M1)	55 (13.9%)	54 (17.3%)
Overall survival (OS)	Survival days (Mean ± SD ^1^)	810.5 ± 833.8	1310.3 ± 1062.7
	Uncensored patients	173 (43.7%)	102 (32.8%)
	Censored patients	223 (56.3%)	209 (67.2%)

^1^ SD: Standard Deviation.

### 2.3. Graph Neural Networks for Survival Prediction

In this section, we detail our proposed architecture of Graph Neural Networks (GNNs), termed GNN-surv, tailored specifically for survival models. Our approach exploits GNNs on a patient similarity graph G to discern and learn from their correlational structures, which depict genomic and clinical similarities among patients. We employ an adjacency matrix *A* of the similarity graph G and a feature matrix *X* to train the GNN model. This model is designed to predict the discrete survival time while acknowledging the right-censored observations.

The GNN-surv architecture comprises multiple GNN layers, each succeeded by batch normalization, ReLU activation, and dropout for efficient learning and training. We also use dropout to regularize the model and prevent overfitting. The model is versatile, capable of using different types of GNN layers, such as GCNConv, SAGEConv, and GATConv. In the following section, we outline the functionality of each GNN layer.

#### 2.3.1. Graph Convolutional Networks (GCN)

Introduced by Kipf and Welling [26], Graph Convolutional Networks (GCN) employ a convolution-based strategy that generates node embeddings by learning from the graph structure and node features, efficiently incorporating local neighborhood information into each node’s embedding. Within each layer, nodes gather information from their immediate neighbors, apply a convolution operation on these features using a shared weight matrix, and pass through an activation function. The operation of GCN for a single layer is as follows:

(3) $$h_{v}^{\left(l+1\right)}=\sigma \left(\frac{1}{\sqrt{D_{v}D_{u}}}\sum_{u\in N(v)}W^{\left(l\right)}h_{u}^{\left(l\right)}\right)$$

In this equation, $h_{v}^{\left(l\right)}$ and $h_{v}^{\left(l+1\right)}$ denote the feature vectors of node *v* at layers *l* and l+1, respectively; N(v) is the set of neighboring nodes to node *v*; $D_{v}$ and $D_{u}$ are the degrees of node *v* and its neighboring node *u*, respectively, used for feature aggregation normalization; $W^{\left(l\right)}$ is the shared learnable weight matrix at layer *l*; and σ is an activation function, specifically a Rectified Linear Unit (ReLU) in this study.

#### 2.3.2. GraphSAGE

Designed to generate node embeddings by sampling and aggregating features from a node’s local neighborhood [27], GraphSAGE can operate on large graphs and generate embeddings for unseen nodes by leveraging node attribute information. Within each GraphSAGE layer, nodes aggregate information from their neighbors using various functions, such as mean, pooling, or LSTM, and subsequently use a learnable weight matrix to transform the aggregated information. The operation of a GraphSAGE layer is as follows:

(4) $$h_{v}^{\left(l+1\right)}=\sigma \left(W^{\left(l\right)}\cdot \mathrm{CONCAT}\left(h_{v}^{\left(l\right)},\mathrm{AGGREGATE}{N(v)}^{\left(l\right)}\left(hu^{\left(l\right)}\right)\right)\right)$$

Here, $h_{v}^{\left(l\right)}$ and $h_{v}^{\left(l+1\right)}$ represent the feature vectors of node *v* at layers *l* and l+1, respectively; ${\mathrm{AGGREGATE}}_{N(v)}^{\left(l\right)}$ is an aggregation function that collects and processes features from the node’s neighborhood N(v) at layer *l*; $W^{\left(l\right)}$ is the learnable weight matrix at layer *l*; and σ is an activation function, specifically a Rectified Linear Unit (ReLU).

#### 2.3.3. Graph Attention Networks (GAT)

Graph Attention Networks (GATs) are variants of GCNs, designed to compute node features by weighting the features of neighboring nodes with attention coefficients [28]. The attention mechanism allows the model to focus more on relevant neighbors and less on less significant ones, offering a level of flexibility that is absent in models like GCN or GraphSAGE. Within each GAT layer, each node calculates the attention coefficients with its neighbors, multiplies these coefficients with the neighbors’ features, and subsequently aggregates this information. The operation of GAT for a single layer is as follows:

(5) $$h_{v}^{\left(l+1\right)}=\sigma \left(\sum_{u\in N(v)}\alpha_{vu}W^{\left(l\right)}h_{u}^{\left(l\right)}\right)$$

In this equation, $h_{v}^{\left(l\right)}$ and $h_{v}^{\left(l+1\right)}$ represent the feature vectors of node *v* at layers *l* and l+1, respectively; $\alpha_{vu}$ are the attention coefficients that weigh the importance of node *u*’s features to node *v*; $W^{\left(l\right)}$ is the learnable weight matrix at layer *l*; and σ is an activation function, specifically a LeakyReLU in this case. The attention coefficients $\alpha_{vu}$ are computed using a shared attention mechanism across all edges in the graph.

#### 2.3.4. Discrete-Time Survival Models

We train the GNN-surv to minimize the loss using the ADAM optimizer, which in turn enables the learning of optimal parameters for the prediction task. The loss function and hazards are computed as defined in the discrete-time survival prediction models that we utilize. Specifically, we employ two discrete-time survival models, Logistic Hazard and the Probability Mass Function (PMF) model, both of which are implemented as per the methodology described in [29].

The Logistic Hazard method, first proposed in [15], is a discrete-time survival prediction model that has been enhanced by the capabilities of neural networks [29]. Also known as Partial Logistic Regression [30] or Nnet-survival [16], this method estimates discrete hazards—the probabilities of event occurrence within discrete-time intervals. By optimizing the survival likelihood, this method serves as a potent tool for survival analysis.

Complementarily, the PMF model, as detailed in [29], adopts a discrete-time survival prediction approach as well. It underlies methods such as DeepHit [14] and Multi-Task Logistic Regression (MTLR) [31]. These methods leverage neural networks to maximize the likelihood of right-censored time-to-event data within discrete time. The approach focuses on parameterizing PMF—the probabilities of discrete outcomes—and optimizing the survival likelihood. By probabilistically representing the distribution of event occurrence times, the PMF model provides a detailed understanding of survival analysis. Moreover, its integration with neural networks enables it to handle complex patterns in the data, thereby contributing to robust and reliable survival predictions.

### 2.4. Performance Evaluation

We evaluate the performance of our model using two metrics, namely the time-dependent concordance index ($C^{td}$) [32] and the integrated Brier score (IBS) [33,34]. Both metrics are utilized in accordance with the implementation described in [29].

In this study, we focus on a supervised node-level prediction problem, where the nodes represent cancer patients at risk. The primary objective of this model is to predict discrete-time survival while accounting for the patients’ censoring status. As the discrete-time models necessitate the discretization of continuous survival time, we adopt the discretization scheme suggested in [29]. This scheme corresponds to either equidistant times or equidistant marginal survival probabilities. Moreover, it interpolates the discrete-time predictions, corresponding to either piecewise constant density functions or piecewise constant hazard rates.

The IBS metric accounts for both the discrimination and calibration of the survival estimates and also accommodates censored individuals by weighting the score inversely against the estimated censoring distribution. As the IBS is significantly influenced by the discretization scheme (which in turn depends on the number of output nodes), we compute the IBS over 100 equidistant points between the minimum and maximum observed times in the validation set during model training, as discussed in [29]. Conversely, the $C^{td}$ only evaluates the discriminative capabilities of a method’s predictions. It is informative to examine both metrics as they might indicate a trade-off between well-calibrated estimates and effective discriminative performance [29]. The best survival prediction performance is considered to be when the IBS is the lowest and the $C^{td}$ is the highest.

For the purpose of our study, we randomly divide the entire sample into an 80% training set and a 20% test set. To further optimize and validate model training, we subdivide the training set into an 80% training subset and a 20% validation subset, resulting in a 64% training, 16% validation, and 20% test set. We repeat this process for 50 iterations of random splits and calculate the mean $C^{td}$ and IBS of the total repetitions in the test set. When partitioning the data, we split the entire graph into three separate graphs: the training graph, the validation graph, and the test graph. This division signifies that GNN-surv possesses an inductive learning capacity, an important attribute as it allows the model to generalize and make predictions on unseen data, enhancing the robustness and utility of our model in real-world applications.

## 3. Results

### 3.1. Experimental Setting

In the process of constructing the patient similarity graph *W*, we carefully adjust the hyperparameter μ, as defined in Equation (2), within an empirical range of 0.1 to 1. This adjustment is made in increments of 0.1 due to the observed sensitivity of the model performance to variations in the hyperparameter μ [25]. The number of neighbors *k* within Equation (2) is set to 20 based on [25], indicating that model performance demonstrates relative insensitivity to changes in the number of neighbors. While creating the adjacency matrix *A*, we define a correlation threshold *c* of 0.5, adhering to the widely accepted consensus that correlations beneath this value are generally considered as low [35].

Our proposed model, GNN-surv, employs multiple GNN layers trained to predict discrete-time survival using discrete survival models. We utilize the PyTorch Geometric library (PyG) implementations [36] for each of the GNN layers: GCN, GraphSAGE, and GAT. To demonstrate the effectiveness of the proposed GNN-surv model, we incorporate a vanilla Multi-Layer Perceptron (MLP) layer with discrete survival models, designating this as MLP-surv in our experiments. It should be noted that the MLP layer does not utilize graph-structured data but is trained with a feature matrix $X\in R^{N\times p}$, which represents *p* gene expression and clinical features across *N* samples.

We empirically set certain hyperparameters for both the GNN models and the MLP model. The GNN model consists of a three-layer network with a hidden layer size of 32. We train the model for a maximum of 500 epochs with a learning rate of 0.001, employing an early stopping scheme. In this training process, we incorporate a batch normalization layer and dropout. The batch size is determined to be 256, with the dropout rate set to 0.7 in the experiments. Additionally, we train with a batch of graphs to manage the complexity of graph data and set the batch size of these data to 32. For a fair comparison, we use the same hyperparameters for the MLP model as well.

### 3.2. Hyperparameter Optimization in Graph Construction

**Figure 1:**
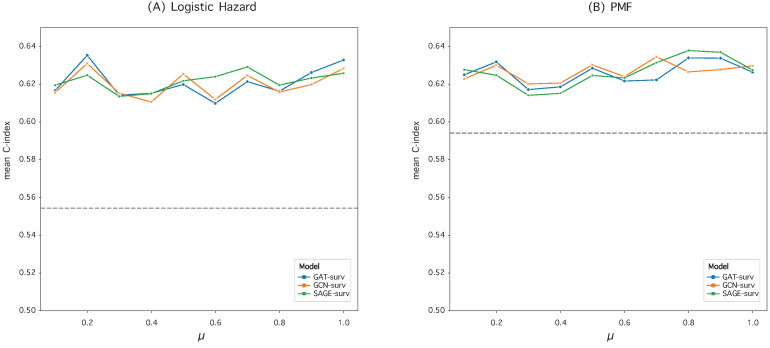
Performance variation of GNN-surv models in the BLCA dataset for different hyperparameter μ values. The discrete-time survival models are (**A**) Logistic Hazard and (**B**) PMF. The performance metrics are mean $C^{td}$ values obtained from 50 random data splits. The gray dotted line represents the mean $C^{td}$ of the MLP model, used as a baseline.

**Figure 2:**
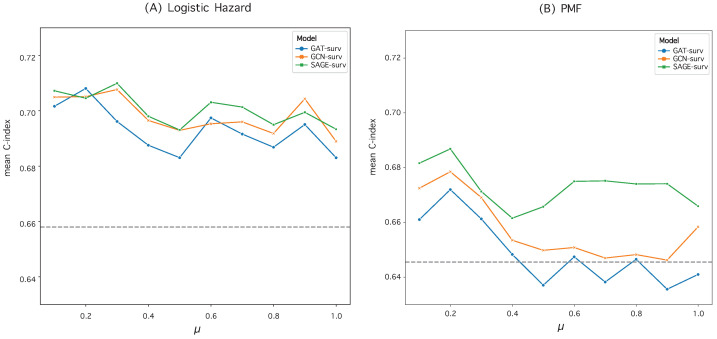
Performance variation of GNN-surv models in the KIRC dataset for different hyperparameter μ values. The discrete-time survival models are (**A**) Logistic Hazard and (**B**) PMF. The performance metrics are mean $C^{td}$ values obtained from 50 random data splits. The gray dotted line represents the mean $C^{td}$ of the MLP model, used as a baseline.

We evaluated and compared the performance of the MLP model and GNN models when paired with two discrete-time survival models: Logistic Hazard and PMF. To optimize the performance, we set the hyperparameter μ empirically during the construction of the patient similarity graph. We conducted an ablation study, detailed in Figure 1 and Figure 2, to find the best μ value. In these studies, the time-dependent concordance index ($C^{td}$) was used as the performance metric.

We trained our GNN-surv models using a similarity network configured with the optimal μ value, which exhibited the best performance for each survival model. Interestingly, the scaling parameter μ demonstrated a negligible impact on the performance of the GNN-surv model in the BLCA dataset, as depicted in Figure 1.

For the KIRC dataset, the Logistic Hazard model maintained robust and enhanced performance. However, when μ was larger, GAT-surv’s performance diminished compared to that of the MLP model in the PMF model, as shown in Figure 2. For this dataset, both GAT-surv and GCN-surv demonstrated relative instability in the PMF model, especially when contrasted with their performance in the BLCA dataset. Nevertheless, SAGE-surv consistently outperformed the MLP model in both survival models, underlining the efficacy of GraphSAGE when implemented in the GNN-surv model in the KIRC dataset.

For the bladder cancer (BLCA) data, we configured the μ parameter to 0.2 for the Logistic Hazard model and to 0.8 for the PMF model. In contrast, for the kidney cancer (KIRC) data, we set μ to 0.3 for the Logistic Hazard model and 0.2 for the PMF model.

### 3.3. Survival Prediction Performance

The assessment of survival prediction performance was executed by evaluating the mean time-dependent concordance index ($C^{td}$) and the integrated Brier score (IBS), along with standard deviations across 50 data splits. The $C^{td}$ is a metric used to evaluate the predictive accuracy of survival models over time. It measures the agreement between predicted and observed survival times, with a higher value indicating better model performance. The IBS quantifies the overall prediction error for a survival model across all time points, with lower values indicating better accuracy and calibration. Detailed results for the bladder cancer (BLCA) and kidney cancer (KIRC) datasets are provided in Table 2 and Table 3, respectively.

The performance comparison of the GNN-surv models and the MLP model within the BLCA and KIRC datasets demonstrates a significant improvement when using GNN-surv models. In the BLCA dataset, the GAT-surv model outperforms the MLP-surv model, with an increase of approximately 14.6% and 7% in the $C^{td}$ metric for the Logistic Hazard and PMF survival models, respectively. Concurrently, the GCN-surv and SAGE-surv models demonstrate superior performance, reducing the IBS by approximately 26.7% and 28.3%, respectively, for the same models. Upon examining the KIRC dataset, the SAGE-surv model emerges as the top performer, achieving a 7.9% and 6.4% increase in $C^{td}$ for the Logistic Hazard and PMF models, respectively, while also reducing the IBS by 24.1% and 8.1%, respectively. Similar performance improvements are also exhibited by the GCN-surv and GAT-surv models.

These results underscore the superior performance of GNN-surv models in survival analysis within these datasets, revealing the benefits of integrating GNN models for discrete survival time-to-event predictions on a patient similarity graph. Given that the MLP model does not account for graph structures, this significant enhancement suggests the existence of a meaningful correlational structure within patient similarity graphs. By effectively utilizing these relationships, the proposed GNN-surv models substantially improve the survival prediction performance.

Upon examining the GNN-surv models, the differential performance between GCN-surv, SAGE-surv, and GAT-surv was relatively small, illustrating the adaptability and resilience of GNN-surv models across different GNN layers. However, a standout observation was the extraordinary performance of the SAGE-surv model within the KIRC dataset. This model demonstrated the highest performance metrics across both survival models, reinforcing the efficacy of the GraphSAGE model in survival prediction applications in the KIRC dataset. Interestingly, the majority of the GNN-surv models portrayed consistent and enhanced performance across both datasets, exhibiting insensitivity to variations in the μ parameter, generally for both survival models. This demonstrates their robustness and effectiveness and suggests that GNN-surv models could be seamlessly adapted to different GNN layers, providing a versatile framework for survival prediction.

**Figure 3:**
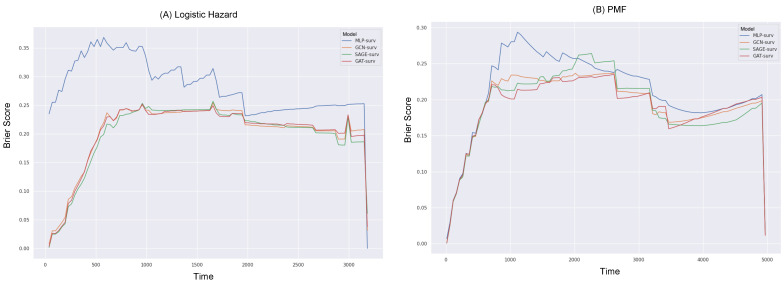
Brier scores over survival time (in days) for different GNN-surv models and MLP model in the BLCA dataset. Discrete-time survival models considered are (**A**) Logistic Hazard and (**B**) PMF.

**Figure 4:**
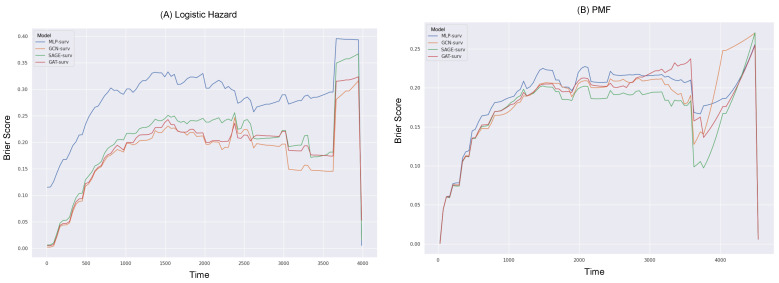
Brier scores over survival time (in days) for different GNN-surv models and MLP model in the KIRC dataset. Discrete-time survival models considered are (**A**) Logistic Hazard and (**B**) PMF.

Furthermore, we delved into the exploration of the Brier scores relative to survival time (in days), as depicted in Figure 3 and Figure 4. For the calculation of the IBS, the Brier score was obtained across 100 equidistant points, falling between the minimum and maximum observed times.

Reiterating the consistent superior performance of the GNN-surv models, these models continued to outperform the MLP model across all durations in the Logistic Hazard model within both datasets. Specifically, the MLP model showed noticeably poor performance during shorter durations across both survival models, in the BLCA data. When the PMF model was utilized, there were instances where SAGE-surv underperformed slightly compared to the MLP model within specific time durations—approximately around median time—in the BLCA data. Furthermore, the performance enhancements of the GNN-surv models, which were previously noted in the Logistic Hazard model, were relatively marginal and displayed instability for larger durations when applied to the PMF model in the KIRC data. However, the majority of the GNN-surv models substantially outperformed the MLP model and demonstrated stable performance across most durations.

The consideration of the integrated Brier score (IBS) revealed that, for both the MLP-surv and GNN-surv models, the PMF model could perform better than the Logistic Hazard. This was an interesting finding, given that no significant differences were observed between the survival models when the time-dependent concordance index ($C^{td}$) was considered. This points to the value of the IBS, as it takes into account both the discrimination and calibration of survival estimates, whereas the Logistic Hazard is primarily concerned with discriminative performance. In other words, the IBS not only considers the model’s discriminative ability but also its calibration. Calibration refers to the accuracy of the model’s predicted probabilities. A well-calibrated model’s predicted probabilities of an event (such as survival in a certain time period) should match the actual proportion of that event in the observed data. In this context, the fact that the PMF model performs significantly better than the Logistic Hazard model when evaluated with the IBS (but not when evaluated with $C^{td}$) suggests that the PMF model’s predicted probabilities may be more accurate (i.e., better calibrated) than those of the Logistic Hazard model.

## 4. Discussion

This study examined the potential of Graph Neural Networks (GNNs), specifically designed to enhance patient survival predictions for urologic cancers (GNN-surv). However, the research was limited by several key limitations. The scope of our investigation was limited to two types of urologic cancers, dictated by the need for a sufficient number of samples and balanced censoring rates. However, the potential to extend our method to a pan-cancer integrative analysis warrants further discussion. Our model’s promising performance in predicting discrete survival outcomes for two urologic cancers hints at the possibility that it could be applicable across a wider range of cancer types. The foundation for such broad application lies in the inherent flexibility of the GNN model, which can adapt to different data types and structures.

Furthermore, the absence of feature selection in our analysis introduced potential challenges. Typically, survival prediction models involve feature selection to focus on statistically significant genes that correlate with survival outcomes. However, in this proof-of-concept study, we excluded feature selection to concentrate on the comparative effectiveness of the GNN model against a simple neural network model. This resulted in the inclusion of an excessive number of redundant genomic features, derived from over 20,000 genes, which may have impacted the overall performance of our models. Despite these limitations, our research provided several noteworthy findings. The superiority of the GNN-surv models over the MLP-surv model indicates that the former’s ability to leverage the correlational structures within patient similarity graphs for survival prediction is beneficial. Furthermore, the enhanced performance of GNN-surv models, even with a excessive number of features and a relatively small sample size, suggests the potential to mitigate the effect of redundant features and extract meaningful patterns in a patient similarity graph for discrete survival prediction.

Despite the demonstrated efficacy of the discrete-time survival models employed in this study, there is potential for further enhancement through the incorporation of continuous-time survival models, such as the Cox Partial Hazard (Cox-PH) model, into GNN-surv models. This could allow for the consideration of time-varying covariates, providing a better understanding of patient survival probabilities over time. Furthermore, this integration could potentially enable the GNN-surv model to capture more intricate temporal dynamics within the data. This is particularly beneficial when dealing with longitudinal data or when a fine-grained temporal resolution is critical for clinical decision making. Moreover, by fusing both discrete- and continuous-time models, the GNN-surv framework could potentially serve as a unified platform for survival prediction, capable of handling a wide spectrum of clinical scenarios and data types.

Lastly, GNNs promise to revolutionize survival prediction models but face limitations, including reliance on robust graph structures and vulnerability to high-dimensionality in data. These challenges arise from the need for well-defined graph structures in the face of complex oncological data and high feature-to-sample ratios, common in biomedical datasets. Bypassing feature selection, GNNs can, however, prioritize informative structures over less significant ones. Thus, the elaborated graph construction methodology also presents an avenue for further exploration. Networks established using diverse types of omics profiles might capture the inherent patient heterogeneity more accurately, enhancing the precision of survival predictions. Future directions to address these constraints include structure learning to optimize graph structures, the development of explainable GNN models for comprehensive patient interaction analysis, and investigating feature-wise interactions such as gene–gene and gene–disease interactions to identify critical biomarkers for survival prediction. Moreover, the integration of other data modalities, such as pathological images, could provide a richer context to these networks, offering a more comprehensive picture of patients’ health conditions.

## 5. Conclusions

To summarize, our research underscores the promising potential of GNN-surv models within the context of discrete-time survival prediction and patient similarity networks. Our findings reveal that GNN-surv models consistently outperformed traditional MLP models across various performance metrics in two urologic cancer datasets. The superior performance of these models could greatly assist clinicians by providing more accurate survival predictions, consequently guiding the formulation of personalized treatment strategies. The successful use of patient similarity graphs in our GNN-surv models also suggests the existence of valuable correlational structures within these networks, offering potential leverage for survival prediction. Despite the aforementioned limitations, our findings signal the potential for the wide applicability of GNN-surv models in survival prediction tasks. The continued exploration and refinement of these models, their application to diverse datasets, and the integration of various survival models could significantly enhance personalized treatment strategies in the realm of oncology research.

## Figures and Tables

**Table 2:** Performance comparison of GNN-surv models and MLP model within the BLCA dataset using two discrete-time survival models, Logistic Hazard and PMF. The metrics utilized for performance assessment include the mean $C^{td}$ and IBS, along with their respective standard deviations, acquired from 50 random data splits. The highest performance for each metric and survival model is highlighted in bold text.

	Logistic Hazard (μ=0.2)		PMF (μ=0.8)	
Model	$C^{\mathrm{td}}$	IBS	$C^{\mathrm{td}}$	IBS
MLP-surv	0.5543 ± 0.0689	0.3183 ± 0.0497	0.5941 ± 0.0629	0.2324 ± 0.0222
GCN-surv	0.6309 ± 0.0481	**0.2331 ± 0.0358**	0.6265 ± 0.0493	**0.2130 ± 0.0231**
SAGE-surv	0.6247 ± 0.0505	**0.2331 ± 0.0389**	**0.6378 ± 0.0415**	0.2140 ± 0.0238
GAT-surv	**0.6352 ± 0.0520**	0.2341 ± 0.0365	0.6339 ± 0.0451	0.2154 ± 0.0229

**Table 3:** Performance comparison of GNN-surv models and MLP model within the KIRC dataset using two discrete-time survival models, Logistic Hazard and PMF. The metrics utilized for performance assessment include the mean $C^{td}$ and IBS, along with their respective standard deviations, acquired from 50 random data splits. The highest performance for each metric and survival model is highlighted in bold text.

	Logistic Hazard (μ=0.3)		PMF (μ=0.2)	
Model	$C^{\mathrm{td}}$	IBS	$C^{\mathrm{td}}$	IBS
MLP-surv	0.6581 ± 0.0559	0.2577 ± 0.0902	0.6455 ± 0.0516	0.2022 ± 0.0174
GCN-surv	0.7077 ± 0.0373	0.1965 ± 0.0240	0.6785 ± 0.0464	0.1964 ± 0.0195
SAGE-surv	**0.7099 ± 0.0409**	**0.1955 ± 0.0269**	**0.6868 ± 0.047**	**0.1859 ± 0.0222**
GAT-surv	0.6962 ± 0.0362	0.2018 ± 0.0260	0.672 ± 0.05	0.1958 ± 0.0233

## Data Availability

The data used in this study come from the publicly accessible TCGA dataset and can be downloaded from UCSC Xena at: http://xena.ucsc.edu.
